# Suppression of FOXM1 Transcriptional Activities via a Single-Stranded DNA Aptamer Generated by SELEX

**DOI:** 10.1038/srep45377

**Published:** 2017-03-30

**Authors:** Qin Xiang, Guixiang Tan, Xia Jiang, Kuangpei Wu, Weihong Tan, Yongjun Tan

**Affiliations:** 1 State Key Laboratory of Chemo/Biosensing and Chemometrics, College of Biology, Collaborative Innovation Center for Chemistry and Molecular Medicine, Hunan University, Changsha, Hunan 410082, China

## Abstract

The transcription factor FOXM1 binds to its consensus sequence at promoters through its DNA binding domain (DBD) and activates proliferation-associated genes. The aberrant overexpression of FOXM1 correlates with tumorigenesis and progression of many cancers. Inhibiting FOXM1 transcriptional activities is proposed as a potential therapeutic strategy for cancer treatment. In this study, we obtained a FOXM1-specific single stranded DNA aptamer (FOXM1 Apt) by SELEX with a recombinant FOXM1 DBD protein as the target of selection. The binding of FOXM1 Apt to FOXM1 proteins were confirmed with electrophoretic mobility shift assays (EMSAs) and fluorescence polarization (FP) assays. Phosphorthioate-modified FOXM1 Apt (M-FOXM1 Apt) bound to FOXM1 as wild type FOXM1 Apt, and co-localized with FOXM1 in nucleus. M-FOXM1-Apt abolished the binding of FOXM1 on its consensus binding sites and suppressed FOXM1 transcriptional activities. Compared with the RNA interference of FOXM1 in cancer cells, M-FOXM1 Apt repressed cell proliferation and the expression of FOXM1 target genes without changing FOXM1 levels. Our results suggest that the obtained FOXM1 Apt could be used as a probe for FOXM1 detection and an inhibitor of FOXM1 transcriptional functions in cancer cells at the same time, providing a potential reagent for cancer diagnosis and treatment in the future.

FOXM1 is a member of the fork head/winged-helix (FOX) transcription factor family and at first is found to activate a network of proliferation-associated genes critical to DNA replication and mitosis^1^,^2^. FOXM1 is frequently overexpressed in multiple cancers through the activation of specific transcriptional pathways^3^. As a consequence, FOXM1 is linked with many tumorigenesis processes, such as oxidative stress response^4^, DNA damage repair^5^, cancer stem cell proliferation^6^, and metastasis^7^,^8^. The inhibition of FOXM1 expression or its activities abolishes the tumorigenesis and is suggested as a novel therapeutic strategy in cancer treatments^9^,^10^,^11^,^12^,^13^,^14^. FOXM1 activates gene transcription processes through its binding to its target gene promoters. Like the other members of FOX family, FOXM1 contains a highly conserved 100-aa DNA binding domain (DBD), which binds to a consensus DNA sequence containing a canonical FKH motif (RYAAAYA) in the promoters of its target genes^15^. Therefore, the attenuation of the DNA binding ability of FOXM1 could suppress FOXM1 transcriptional activities and represent a potent strategy targeting FOXM1 in cancer cells^16^.

Aptamers are single-stranded oligonucleotides obtained from a combinatorial oligonucleotide library through a method referred to the systematic evolution of ligands by exponential enrichment (SELEX)^17^,^18^. The selected single-stranded oligonucleotides are capable of specific and high-affinity binding to target molecules due to their tertiary structures^19^. Compared with conventional antibodies, aptamers recognize their target molecules with a number of attractive features, including low molecular weight, quick and reproducible synthesis *in vitro*, easy modification, good stability, low toxicity, low immunogenicity, and rapid tissue penetration^20^. The aptamers selected directly against proteins can interfere with their target protein activities and are good tools to be used to investigate the mechanisms of protein-protein and/or protein-nucleic acids interaction. These advantages have also made aptamers excellent alternatives for diagnostic and/or therapeutic purposes in disease treatment^21^.

So far, several small-molecule inhibitors of FOXM1 have been obtained but none of them are good enough for clinical usage^9^,^10^,^14^, requiring the development of novel FOXM1 inhibitors. In this study, we obtained a FOXM1-specific aptamer (FOXM1 Apt) targeting the FOXM1 DBD. Phosphorthioate-modified FOXM1 Apt (M-FOXM1 Apt) possessed a similar binding ability to FOXM1 as FOXM1 Apt, and was able to prevent FOXM1 binding to the promoters of its target genes, followed by the suppression of FOXM1 transcriptional activities. The transfection of M-FOXM1 Apt resulted in a dramatic inhibition of cell proliferation and down-regulation of the expression of FOXM1 target genes without changing the levels of FOXM1 mRNA and protein in cancer cells. The obtained FOXM1-Apt could be used as a probe for FOXM1 detection and an inhibitor of FOXM1 transcriptional function in cancer cells at the same time, providing a potential reagent for cancer diagnosis and therapy in the future.

## Results

### The selected FOXM1 Apt from SELEX bound to FOXM1 DBD and full-length protein

**Figure 1:**
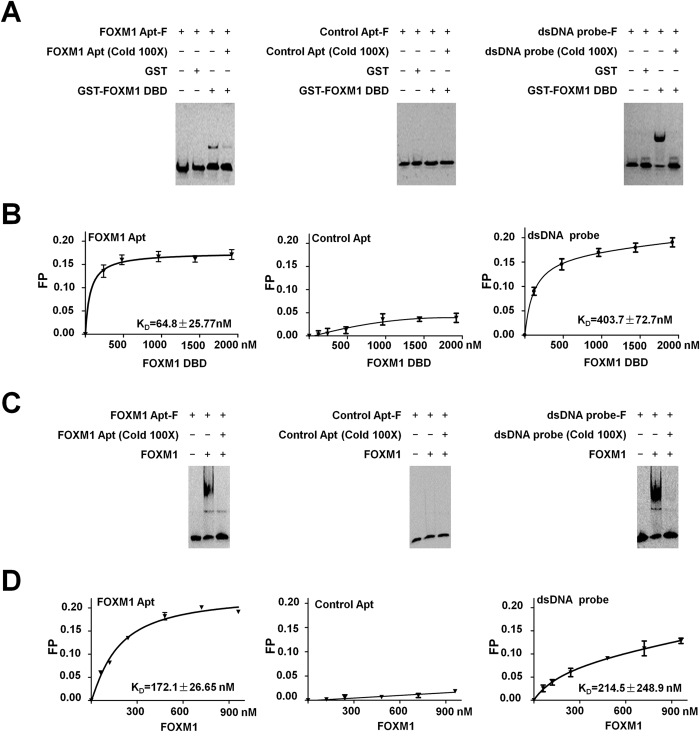
The obtained FOXM1 Apt bound to FOXM1 DBD and full-length protein. (**A**) The FOXM1 Apt bound to FOXM1 DBD specifically in the EMSA experiment. The ROX-labeled FOXM1 Apt (FOXM1 Apt-F) (50 nM) was used for EMSAs with the purified GST or GST-FOXM1 DBD fusion proteins (0.5 mg/ml). The unlabeled FOXM1 Apt (100x) was added to the reaction to show specificity of FOXM1 Apt/GST-FOXM1 DBD complex formation. The ROX-labeled Control Apt (Control Apt-F) (50 nM) or the FAM-labeled double strand DNA probe containing FOXM1 putative binding sequence (dsDNA probe-F) (50 nM) was also used for EMSAs with GST or GST-FOXM1 DBD fusion proteins as the negative or positive controls respectively in the assays. (**B**) Detection of the binding affinity between FOXM1 Apt and FOXM1 DBD. The fluorescence polarization (FP) assays were performed with FOXM1 Apt-F, Control Apt-F, or dsDNA probe-F (10 nM each sample) plus the FOXM1 DBD protein at different concentration (0, 120, 240, 960, 1440, 1920 nM). The FP values were obtained with the EX535 nm/EM600 nm filter for ROX or the EX405 nm/EM535 nm filter for FAM in Fluoromax-4NIR (Horiba). (**C**,**D**) Identification of the binding ability and affinity of FOXM1 Apt to FOXM1 full-length protein. FOXM1 Apt-F (50 nM) was used for EMSA experiments with the purified His-FOXM1 full-length protein (FOXM1) (0.5 mg/ml). The unlabeled FOXM1 Apt (100x) was added to the reaction to show specificity of FOXM1 Apt/FOXM1 complex formation. Control Apt-F (50 nM) or dsDNA probe-F (50 nM) was also used for EMSAs with His-FOXM1 full-length protein as the negative or positive controls respectively in the assays (**C**). The FP assays were performed with FOXM1 Apt-F, Control Apt-F, or dsDNA probe-F (50 nM) plus the His-FOXM1 protein at different concentration (0, 240, 480, 720, 960 nM). The FP values were obtained according to the procedure described above. Data shown are the mean values of three assay points (±S.E.M.).

To obtain FOXM1-specific aptamers, we purified Glutathione S-transferase (GST) and GST-FOXM1 DBD (225–355 aa) fusion proteins through an *E. coli* protein expression system as the negative or positive target molecules for SELEX (Fig. S1A,B). The FOXM1 DBD-specific aptamers were selected from a carboxy-X-rhodamine (ROX)-labeled ssDNA library according to the SELEX procedure (Fig. S1C). After 6 rounds of SELEX, the enriched aptamers were confirmed to bind to FOXM1 DBD by incubated with GST-FOXM1 DBD fusion protein-Glutathione Sepharose beads and observed with fluorescence microscope (Fig. S1D). These enriched ssDNAs of the sixth round of SELEX were amplified, cloned, and sequenced. From the multiple cloned sequences (see Table 1), we selected the most abundant sequence (Clone 1, C1) (5′-TCA CTT CAC ACC GCA TCT CTA CGT CCG GTT GCG CTT TCC TTT-3′) and named it as FOXM1 Apt for further studies. The mutated FOXM1 Apt (5′- TCA CTT AAA AAA AAA AAA CTC ATG AAA AAA AAA AAA TCC TTT-3′) was also generated as the control aptamer (Control Apt) in following experiments. The Electrophoretic Mobility Shift Assays (EMSAs) were performed with ROX-labeled FOXM1 Apt (FOXM1 Apt-F) and the purified GST-FOXM1 DBD fusion proteins. FOXM1 Apt-F formed a DNA/protein complex in EMSAs with FOXM1 DBD and the addition of an unlabeled FOXM1 Apt (100x) disturbed the formation of the DNA/protein complex, while the ROX-labeled Control Apt (Control Apt-F) could not form the FOXM1 DBD/DNA complex, confirming the specific binding of FOXM1 Apt to FOXM1 DBD (Fig. 1A). The FAM-labeled double strand DNA probe containing FOXM1 putative binding sequence (dsDNA probe-F) was also used for EMSAs with GST or GST-FOXM1 DBD fusion proteins as positive controls in the assays (Fig. 1A). In order to detect the binding affinity between FOXM1 Apt and FOXM1 DBD, the fluorescence polarization (FP) assays were performed with FOXM1 Apt-F, Control Apt-F, or dsDNA probe-F plus the FOXM1 DBD proteins. A published result found that FOXM1 DBD had a micromolar affinity for the classical Forkhead consensus site^15^. As predicted, the dissociation constant (K_D_) of the dsDNA probe to FOXM1 DBD was 542.8 ± 147.9 nM. The K_D_ of FOXM1 Apt for FOXM1 DBD was 64.8 ± 25.77 nM, which was at least an order of magnitude lower than that of the dsDNA probe (Fig. 1B), suggesting a stronger binding of FOXM1 Apt than that of the dsDNA probe to FOXM1 DBD. We also tested the binding abilities of several other cloned sequences to FOXM1 DBD with EMSAs and FP assays. Although the tested sequences could bind to FOXM1 DBD in EMSAs, they all showed weaker affinities than FOXM1 Apt to FOXM1 DBD in FP assays (Fig. S2). Furthermore, a His-FOXM1 full-length protein was purified (Fig. S3) and used to identify the binding ability of FOXM1 Apt to FOXM1 full-length protein. FOXM1 Apt was found to interact with FOXM1 full length protein by EMSAs (Fig. 1C). The FP assays determined that the K_D_ of FOXM1 Apt-F to FOXM1 full-length protein was 172.1 ± 26.55 nM, lower than that of the dsDNA probe (214.5 ± 248.9 nM), indicating that FOXM1 Apt could specifically bind to FOXM1 full length protein with a high affinity (Fig. 1D).

### The phosphorthioate-modified FOXM1 Apt (M-FOXM1 Apt) maintained the binding activities to FOXM1 protein

**Figure 2:**
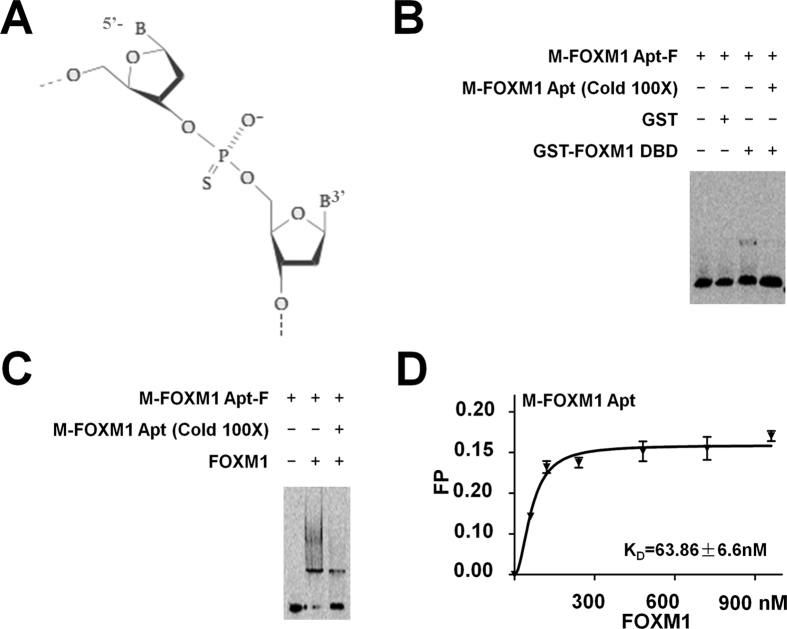
The phosphorthioate-modified FOXM1 Apt (M-FOXM1 Apt) bound to FOXM1 protein. (**A**) The schematic of phosphorthioate modification. The internucleotide linkages of FOXM1 Apt can be modified by the sulfur substitution in the phosphate ester backbone to form phosphorthioate linkages. (**B**) M-FOXM1 Apt bound to FOXM1 DBD specifically in the EMSA experiment. The ROX-labeled M-FOXM1 Apt (M-FOXM1 Apt-F) (50 nM) was used for EMSAs with the purified GST or GST-FOXM1 DBD fusion proteins (0.5 mg/ml). The unlabeled M-FOXM1 Apt (100x) was added to the reaction to show specificity of FOXM1 Apt/GST-FOXM1 DBD complex formation. (**C**,**D**) Identification of the binding ability and affinity of M-FOXM1 Apt to FOXM1 full-length protein. M-FOXM1 Apt-F (50 nM) was used for EMSAs with the purified His-FOXM1 full-length protein (FOXM1) (0.5 mg/ml). The unlabeled M-FOXM1 Apt (100x) was added to the reaction to show specificity of FOXM1 Apt/FOXM1 complex formation (**C**). The FP assays were performed with M-FOXM1 Apt-F (10 nM each sample) plus the His-FOXM1 protein at different concentration (0, 240, 480, 720, 960 nM). The FP values were obtained with the EX535 nm/EM600 nm filter for ROX (**D**). Data shown are the mean values of three assay points (±S.E.M.).

Wild-type DNA aptamers are too susceptible to nuclease-mediated degradation to be useful for *in vivo* applications^20^. Among multiple approaches for modifying oligonucleotides, we chose internucleotide phosphorothioate linkage modification^22^ for FOXM1 Apt. The internucleotide linkages of FOXM1 Apt were modified by the sulfur substitution in the phosphate ester backbone to form phosphorothioate linkages (Fig. 2A). As the consequence of phosphorothioate modification, the molecular mass of the modified FOXM1 Apt (M-FOXM1 Apt) was elevated in the measurement of mass spectrometry (Fig. S4A). The M-FOXM1 Apt possessed a similar spatial structure as the FOXM1 Apt, evidenced by the Circular Dichroism (CD) measurement in the parallel structure formation for the FOXM1 Apt and M-FOXM1 Apt, although M-FOXM1 Apt showed enhanced negative peaks at 211 nm and 248 nm and an elevated positive peak at 277 nm (Fig. S4B). M-FOXM1 Apt obtained an enhanced stability in the DNase I treatment compared to FOXM1 Apt (Fig. S4C). The EMSA experiments were performed with ROX-labeled M-FOXM1 Apt (M-FOXM1 Apt-F) and FOXM1 DBD or FOXM1 full-length protein, and confirmed that M-FOXM1 Apt maintained the binding activities to FOXM1 protein (Fig. 2B,C). The FP assays determined that the K_D_ of M-FOXM1 Apt-F to FOXM1 full-length protein was 63.86 ± 6.6 nM (Fig. 2D), implicating that the phosphorthioate modification in FOXM1 Apt even improved its binding affinity to FOXM1 proteins compared to wild-type FOXM1 Apt (FOXM1 Apt to FOXM1 protein: K_D_ = 172.1 ± 26.55 nM, see above). Therefore, we used M-FOXM1 Apt-F for further experiments.

### M-FOXM1 Apt bound to FOXM1 protein specifically in cancer cells

**Figure 3:**
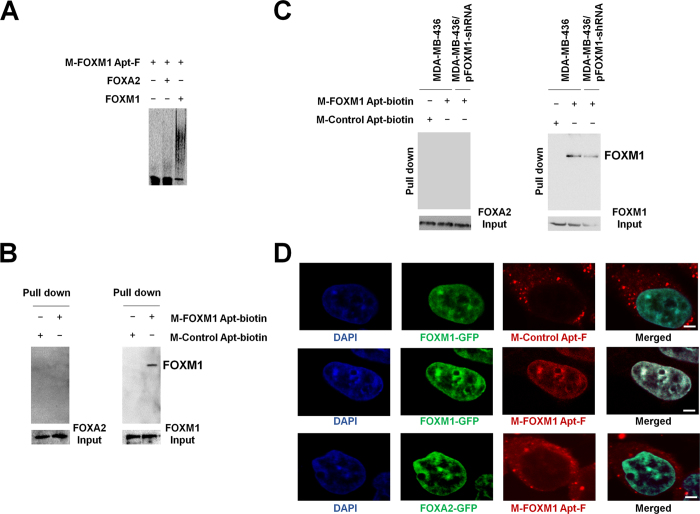
The binding specificity of M-FOXM1 Apt to FOXM1 protein. (**A**) M-FOXM1 Apt bound to FOXM1 protein but not FOXA2 protein in EMSAs. M-FOXM1 Apt-F (50 nM) was used for EMSA experiments with the purified His-FOXA2 full-length protein (FOXA2) or His-FOXM1 full-length protein (FOXM1) (0.5 mg/ml). Electrophoresis was performed and M-FOXM1 Apt/protein complexes were visualized by fluorescence imaging. (**B**) M-FOXM1 Apt bound to FOXM1 protein but not FOXA2 protein in Pull-down assays. Biotinylated M-FOXM1 Apt (M-FOXM1 Apt-biotin) (5 μM final concentration) were incubated with lysates of pCMV-FOXA2-transfected or pCMV-FOXM1-transfected HEK293T cells. Streptavidin agarose beads were added to pull down M-FOXM1 Apt-biotin/protein complexes, which were detected by Western Blotting with anti-FOXA2 or anti-FOXM1 antibody. Biotinylated M-Control Apt (M-Control Apt-biotin) was used as negative controls in the Pull-down experiments. One percent of input of lysates (500 μg) was also used for Western Blotting as positive loading controls. (**C**) M-FOXM1 Apt bound to FOXM1 protein but not FOXA2 protein in MDA-MB-436 breast cancer cells. The lysates (500 μg) of MDA-MB-436 cells were used to repeat the Pull-down assays according to the procedure described above. In addition, pFOXM1-shRNA plasmids (300 nM) were transfected into MDA-MB-436 cells and 48 hr later the lysates were prepared for the Pull-down experiment with M-FOXM1 Apt-biotin. One percent of input of lysates (500 μg) was also used for Western Blotting as positive loading controls. (**D**) M-FOXM1 Apt was co-localized with FOXM1 proteins in cells. HEK293T cells were transfected with pCMV-FOXM1-GFP or pCMV-FOXA2-GFP (5 μg) and 24 hr later were transfected with M-FOXM1 Apt-F (1 μM) or M-Control Apt-F (1 μM) separately. The localization of FOXM1-GFP or FOXA2-GFP and the ROX-labeled aptamers (M-FOXM1 Apt-F or M-Control Apt-F) was imaged with the fluorescent Con-focal microscope (Olympus FluoView FV1000) (1000x). DAPI staining showed the location of nuclei of cells. (Scale bars: 5 μm).

There are more than 50 members identified in FOX transcription factor family and all of them contain a highly conserved DBD. Because FOXM1 Apt was selected by using FOXM1 DBD as the target molecule in SELEX, we had to determine whether the binding of FOXM1 Apt to FOXM1 was highly specific. We tested whether or not M-FOXM1 Apt was able to bind to other FOX proteins such as FOXA2, which is the first cloned mammalian FOX transcription factor containing the typical conserved DBD^23^. A His-FOXA2 full-length protein was purified (Fig. S5) and used for EMSAs with M-FOXM1 Apt. In the same experimental condition, M-FOXM1 Apt bound to FOXM1 but not FOXA2 (Fig. 3A). To further test that FOXM1 Apt could recognize FOXM1 specifically from a mixture of cellular proteins, we first prepared whole-cell lysates from the FOXM1-overexpressing cells in which FOXM1 expression plasmid vectors were transfected (Fig. S6A). As a control, the lysate of the FOXA2-overexpressing cells was also prepared (Fig. S6B). Biotinylated M-FOXM1 Apt (M-FOXM1 Apt-biotin) was generated and incubated with FOXM1-overexpressing or FOXA2-overexpressing cell lysates. Streptavidin agarose beads were added to the reactions to pull down M-FOXM1 Apt-biotin/protein complexes, which were detected by Western Blotting only in the reaction of M-FOXM1 Apt-biotin plus FOXM1-overexpressing lysates (Fig. 3B), suggesting a specific interaction between FOXM1 Apt and FOXM1 proteins in the complicated conditions. We then prepared lysates from MDA-MB-436 breast cancer cells endogenously expressing both FOXM1 and FOXA2, and repeated the experiments described above to confirm the specific interaction between FOXM1 Apt and FOXM1 proteins in the cancer cells (Fig. 3C). The specificity of the binding of FOXM1 Apt to FOXM1 proteins was further determined by Pull-down experiments with MDA-MB-436 cell lysates in which the endogenous FOXM1 expression was knocked down by the transfection of pFOXM1-shRNA vectors to the cells (Fig. 3C). Furthermore, to confirm the recognition of FOXM1 Apt to FOXM1 proteins in cells, we transfected HEK293T cells with pCMV-FOXM1-GFP plasmids expressing FOXM1-GFP fusion proteins, followed by the transfection of M-FOXM1 Apt-F one day later. Observed under a fluorescent Con-focal microscope, FOXM1-GFP fusion proteins localized in nuclei of cells (Fig. 3D). Compared to Control Apt that stayed in the cytosol of cells, FOXM1 Apt was found to co-localize with FOXM1 proteins in nuclei of cells (Fig. 3D), suggesting that FOXM1 Apt could specifically bind to FOXM1 in cells. As a control experiment, HEK293T cells were also transfected with pCMV-FOXA2-GFP plasmids expressing FOXA2-GFP fusion proteins, followed by the transfection of M-FOXM1 Apt-F. We found that the FOXA2-GFP fusion proteins localized in nuclei but FOXM1 Apt stayed in cytoplasm, confirming no interactions between FOXM1 Apt and FOXA2 (Fig. 3D).

### FOXM1 Apt suppressed the transcriptional activities of FOXM1

**Figure 4:**
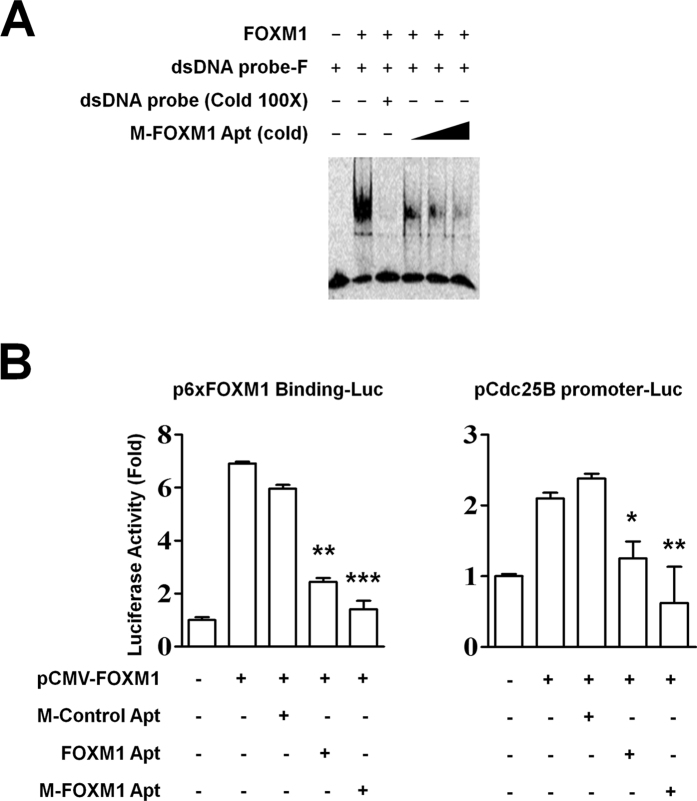
M-FOXM1 Apt suppressed the transcriptional activities of FOXM1. (**A**) M-FOXM1 Apt abolished the binding ability of FOXM1 to its putative DNA binding sequence. The dsDNA probe-F (50 nM each sample) was mixed with His-FOXM1 proteins (0.5 mg/ml) for EMSAs. The M-FOXM1 Apt (unlabeled) was added to the reactions at increased concentration (500, 2500, 5000 nM) to act as the competitor of FOXM1/DNA binding. The unlabeled dsDNA probe (100x) was used to show specificity of FOXM1/DNA complex formation. (**B**) M-FOXM1 Apt prevented FOXM1 transcriptional activities. The FOXM1 expression vector pCMV-FOXM1 (1 μg) was cotransfected with a luciferase reporter plasmid containing 6x FOXM1 binding sequences in its promoter (p6xFOXM1 Binding-Luc, 1 μg) or a −2.3 kb Cdc25B promoter-luciferase reporter plasmid (pCdc25B promoter-Luc, 1 μg) plus loading control pRL-CMV luciferase reporter plasmid (20 ng) into HEK293T cells. M-Control Apt, FOXM1 Apt, or M-FOXM1 Apt (500 nM each sample) was added to the selected transfection. Protein lysates were prepared at 48 hr after transfection and used for the measurement of dual Luciferase activities. Data shown are the mean values of three assay points (±S.E.M.). The asterisks indicate statistically significant changes to the positive control sample (pCMV-FOXM1 only): *P ≤ 0.05, **P ≤ 0.01, ***P ≤ 0.001.

FOXM1 is linked to various types of human malignancies through stimulating the expression of cancer cell-related target genes^3^. The transcriptional activation of FOXM1′s target genes requires FOXM1 binding to its target gene promoters^15^. To test whether FOXM1 Apt could affect FOXM1 transcriptional activities, we first performed EMSAs and determined that M-FOXM1 Apt abolished the binding ability of FOXM1 to its putative DNA binding sequence dramatically *in vitro*, at a dose-dependent manner (Fig. 4A). The wild type FOXM1 Apt also showed similar inhibitory effects on the FOXM1-DNA binding ability as M-FOXM1 Apt (Fig. S7). Based on previous studies^15^,^24^, the cotransfection of FOXM1 expression vector in cells could stimulate the promoter activities of the luciferase reporter plasmids, whose luciferase expression was controlled by either an artificial 6x FOXM1 binding sequence-containing promoter or an endogenous −2.3 kb promoter of Cdc25B (FOXM1′s target gene) (Fig. 4B). The addition of FOXM1 Apt or M-FOXM1 Apt to the cotransfection inhibited the FOXM1-mediated stimulation on the promoter activities significantly while M-FOXM1 Apt appeared better effects than the wild type FOXM1 Apt (Fig. 4B), demonstrating that FOXM1 Apt was able to prevent FOXM1 transcriptional activities *in vivo*. The control aptamer M-Control Apt did not affect FOXM1 transcriptional activities in the cells (Fig. 4B).

### FOXM1 Apt suppressed cell growth through inhibiting the expression of proliferation-related target genes of FOXM1

**Figure 5:**
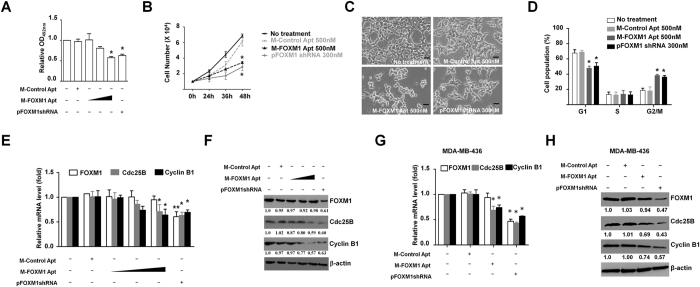
M-FOXM1 Apt suppressed cell proliferation through inhibiting the expression of proliferation-related target genes of FOXM1. (**A**) The 293T-FOXM1 cells (1 × 10^3^ cells/well in triplicates) were seeded in 96-well plates for 12 hr and transfected with M-Control Apt (500 nM), M-FOXM1 Apt (100 nM, 300 nM, 500 nM), or pFOXM1shRNA (300 nM). MTT measurements were performed 48 hr later. (**B**) The 293T-FOXM1 cells (1 × 10^4^ cells/well in triplicates) were seeded in six-well plates for 12 hr and transfected with M-Control Apt (500 nM), M-FOXM1 Apt (500 nM), or pFOXM1shRNA (300 nM). The cell numbers in each well were counted at different time points (24 hr, 36 hr, 48 hr) post transfection. (**C**) The 293T-FOXM1 cells were transfected with M-Control Apt (500 nM), M-FOXM1 Apt (500 nM), or pFOXM1shRNA (300 nM) and 48 hr later the cells were imaged with the microscope (TE-2500, NIKON) (100x). (Scale bars: 50 μm). (**D**) The 293T-FOXM1 cells were transfected with M-Control Apt (500 nM), M-FOXM1 Apt (500 nM), or pFOXM1shRNA (300 nM) in triplicates and 48 hr later the cells were collected, stained with propidium iodide, and analyzed for DNA content on a QuantaSC flow cytometer (Beckman). (**E**,**F**) The 293T-FOXM1 cells were transfected with M-Control Apt (500 nM), M-FOXM1 Apt (100 nM, 300 nM, 500 nM), or pFOXM1shRNA (300 nM) and 48 hr later the cell samples were harvested for the preparation of total RNA and total proteins. The levels of FOXM1, Cdc25B, and Cyclin B1 in the cells were examined by qPCR for mRNA levels (**E**) and by Western blotting for protein levels (**F**). (**G**,**H**) MDA-MB-436 breast cancer cells were transfected with M-Control Apt (500 nM), M-FOXM1 Apt (500 nM), or pFOXM1shRNA (300 nM) and 48 hr later the cell samples were harvested for the preparation of total RNA and total proteins. The levels of FOXM1, Cdc25B, and Cyclin B1 in the cells were examined by qPCR for mRNA levels (**G**) and by Western blotting for protein levels (**H**). Data shown are the mean values of three assay points (±S.E.M.). The asterisks indicate statistically significant changes to the M-Control Apt-treated control sample: *P ≤ 0.05 and **P ≤ 0.01.

To test whether FOXM1 Apt could potentially disturb the proliferation of cancer cells through abolishing FOXM1 transcriptional activities, we first generated a cancer cell line stably overexpressing FOXM1 (293T-FOXM1 cells), in which the consequences of the loss-function of FOXM1 were easily observed. The transfection of M-FOXM1 Apt to 293T-FOXM1 cells resulted in the decrease of cell viabilities at a dose-dependent manner by MTT analysis (Fig. 5A). Interestingly, the transfection of pFOXM1-shRNA vectors expressing FOXM1-specific interference shRNA to the cells also caused similar effects (Fig. 5A), implicating that the ability of M-FOXM1 Apt disturbing the functions of FOXM1 was as powerful as that of the FOXM1 RNA interference. The results of the growth curve and morphological changes from M-FOXM1 Apt-treated 293T-FOXM1 cells also supported this conclusion (Fig. 5B,C). Flow cytometry was used to analyze cell cycle and further showed the disturbed cell proliferation in M-FOXM1 Apt-treated cells (Fig. 5D). To test whether FOXM1 Apt affected the expression of FOXM1 and its target genes, we measured the levels of the mRNA and protein for FOXM1, Cdc25B, and Cyclin B1, among which the last two are FOXM1-stimulated genes^24^,^25^, at the 48 hr time point post the M-FOXM1 Apt treatment. As predicted, the transfection of pFOXM1-shRNA vectors in 293T-FOXM1 cells resulted in the down-regulation of the expression of FOXM1 target genes Cdc25B and Cyclin B1, correlated with the knockdown of FOXM1 mRNA and protein levels (Fig. 5E,F). On the other hand, the treatment of M-FOXM1 Apt at the high dosage in 293T-FOXM1 cells also caused a dramatic down-regulation of the expression of Cdc25B and Cyclin B1 without changing the levels of FOXM1 mRNA and protein in the cells (Fig. 5E,F), implicating that the inhibitory effects of FOXM1 Apt on FOXM1 resulted from disturbing FOXM1 transcriptional activation function but not from affecting FOXM1 expression. As a control, we also tested whether the proliferation of wild type 293T cells with a very low endogenous FOXM1 expression was affected by FOXM1 Apt. The M-FOXM1 Apt treatment at the dosage (500 nM) that abolished the proliferation of 293T-FOXM1 cells did not show dramatic effects on 293T cells (Fig. S8). In addition, we treated MDA-MB-436 breast cancer cells with M-FOXM1 Apt and measured the levels of FOXM1, Cdc25B, and Cyclin B1. The treatment of M-FOXM1 Apt caused a dramatic down-regulation of the expression of Cdc25B and Cyclin B1 without obvious changing of FOXM1 levels in MDA-MB-436 cells (Fig. 5G,H). Taken together, these results determined that FOXM1 Apt could suppress the cellular proliferation of cancer cells by abolishing the functions of FOXM1 *in vivo*.

## Discussion

Aptamers have been suggested as diagnostic or therapeutics reagents because of their high affinity and specificity towards selected targets and abolishing the functions of their targets without obvious side-effects^20^. In 2005, FDA approved Macugen, a RNA aptamer targeting VEGF, as the first aptamer therapeutic for age-related macular degeneration or diabetic retinopathy^21^. A number of aptamer-based therapeutics are currently undergoing clinical trials, including therapeutic aptamers for cancer treatment such as AS1411 targeting nucleolin^26^ and NOX-A12 targeting stroma cell-derived factor-1 (SDF-1)^27^. An aptamer specific for modulating the function of intracellular transcription factor NF-κB has been developed and shows effective inhibition of NF-κB *in vitro* and *in vivo*^28^,^29^, suppressing non-small cell lung cancer resistance to Doxorubicin^30^. An aptamer that inhibits the function of the E2F family of transcription factors has also been obtained and can be potentially used for preventing tumor development^31^. In this study, we obtained the FOXM1-specific single stranded DNA aptamer FOXM1 Apt to target FOXM1 DBD and full-length protein specifically. FOXM1 Apt was confirmed to prevent the binding of FOXM1 to its consensus binding sites in promoters and consequently suppress FOXM1 transcriptional activities, resulting in the downregulation of the expression of FOXM1 target genes and the inhibition of cancer cell proliferation.

FOXM1 is ubiquitously expressed in proliferating and regenerating mammalian cells^1^ and is a key cell cycle regulator of both the transition from G1 to S phase and the progression to mitosis by regulating transcription of cell cycle genes^2^. Loss of FOXM1 expression causes diminished DNA replication^32^, mitotic spindle defects^33^, and mitotic catastrophe^34^. Furthermore, we and others have shown that FOXM1 is involved in contra-acting stresses induced by cytotoxic or genotoxic signals, such as oxidative stress or DNA damage, and enhances the drug resistance of cancer cells^35^. Moreover, we have characterized that FOXM1 plays an essential role in maintenance of cell stemness and its expression is absent from differentiated cells^36^,^37^. These observations suggest that FOXM1 is associated with cancer initiation and progression through its critical roles in cell proliferation, drug resistance, and malignant transformation of undifferentiated cells. This notion is apparently supported by the fact that FOXM1 is highly expressed in various types of human malignancies, such as lung cancer^38^, prostate cancer^39^, basal cell carcinomas^40^, hepatocellular carcinoma^41^, gastric cancer^42^, and squamous cell carcinoma^43^, demonstrating FOXM1 as a diagnostic marker for monitoring the initiation and progression of multiple human cancers. The inactivation of FOXM1 leads to inhibition of progression and/or invasion of these cancers, suggesting FOXM1 as an attractive target for the development of novel anti-cancer therapies. Small chemical compounds and FOXM1-specific RNA interference adenovirus vectors has been developed to inhibit FOXM1 functions for cancer treatment^9^,^10^,^11^,^12^,^13^,^14^. It is necessary to explore more FOXM1-targeting therapeutic strategies that would finally reach the stage of clinical usage. Therefore, FOXM1 Apt obtained from this study provided a potential reagent to detect and repress FOXM1 at the same time, good for cancer diagnosis and treatment in the future.

We confirmed the specificity of FOXM1 Apt binding to FOXM1 by showing that the aptamer was not able to bind to FOXA2, which also contains the typical conserved DBD of FOX transcription factors. In addition, we found that FOXM1 Apt could not interact with FOXP2, another member of FOX transcription factors, in the pull-down experiments with FOXM1 Apt and MDA-MB-436 cell lysates (data not shown). To understand how FOXM1 Apt recognizes FOXM1, we predicted the secondary structure of the aptamer that comprises a primarily stem-loop-loop structure (Fig. S9A). We generated three truncated aptamers, FOXM1 Apt (1–15 nt), FOXM1 Apt (11–34 nt) and FOXM1 Apt (28–42 nt), based on the structural motifs of FOXM1 Apt (Fig. S9B). Even though the CD measurement could not tell obvious differences in spatial structures among these truncated aptamers (Fig. S9C), EMSA experiments with FOXM1 full-length protein confirmed that only the truncated FOXM1 Apt (11–34 nt) possessed FOXM1-binding activities but much weaker than that of wild-type FOXM1 Apt (Fig. S9D). This finding suggested that the 11–34 nt sequence of FOXM1 Apt was the core binding sequence mediating the interaction between FOXM1 Apt and FOXM1 protein. Aptamers often bind to functionally important parts of target proteins and affect protein-protein or protein-nucleic acid interaction of the target proteins^20^. It is still a challenge to predict the binding structure of an aptamer recognizing its target protein according to its nucleotide sequence, resulting in that pharmacokinetic and other systemic properties of the aptamer are variable and often hard to be predicted in practice. With the solved crystal structure of FOXM1 DBD and FOXM1 protein^15^, our study provided a suitable pair of molecules FOXM1 Apt and FOXM1 protein to learn the detail mechanisms how FOXM1 Apt interacting with FOXM1. Further research will concentrate on the analysis of FOXM1 Apt-FOXM1 DBD crystal structure, identify the key nucleotide(s) and conformations of FOXM1 Apt to mediate the interaction between the two molecules, and hopefully provide clues to predict or even design aptamers for target proteins in the future.

In general, native ssDNA aptamer molecules are too susceptible to nuclease-mediated degradation to be useful for most therapeutic applications. When unmodified aptamers enter the cell or are administered *in vivo*, they are rapidly degraded by nucleases^20^. Therefore, chemical modifications of the oligonucleotides are often required to increase resistance to degradation by nucleases. Several strategies have been developed to increase the stability of aptamers without compromising the binding affinity and specificity towards their targets. The nucleotides of aptamers can be partially or completely substituted with one or more modifications, including 2′-amino pyrimidines^44^,^45^, 2′-fluoro pyrimidines^46^, and 2′-O-methyl ribose purines and pyrimidines^47^. Internucleotide linkages can also be modified to phosphorothioate linkages^22^ and high molecular mass polyethylene glycol (PEG) can be conjugated to the 5′-terminus^48^. In this study, we chose to modify the internucleotide linkages of FOXM1 Apt to phosphorothioate linkages. The modified M-FOXM1 Apt possessed a similar spatial structure as FOXM1 Apt and obtained an enhanced resistance to nuclease-mediated degradation. M-FOXM1 Apt imparted greater affinities to FOXM1 protein compared to wild-type FOXM1 Apt, consistent to other findings that sulfur substitution of the phosphate ester backbone in aptamers often results in enhanced binding to their target proteins^22^. Whether M-FOXM1 Apt shows better performance to detect and/or repress FOXM1 in animal models than FOXM1 Apt needs be further evaluated.

In conclusion, the obtained FOXM1 Apt from this study could selectively bind to FOXM1 protein, co-localize with FOXM1 in nucleus after entering cells, and potentially abolish the binding abilities of FOXM1 to its target gene promoters. By this way, the transfection of FOXM1 Apt results in the downregulation of the expression of FOXM1 target genes in cells and consequently inhibits cancer cell proliferation. FOXM1 Apt is a novel and specific FOXM1 inhibitor, providing a potential reagent for cancer diagnosis and therapy in the future.

## Methods

### Cell Culture

HEK 293T and MDA-MB-436 cells were obtained from ATCC and HEK 293T-FOXM1 stable cell line was preserved in our laboratory^8^. HEK 293T and HEK 293T-FOXM1 cells were grown in Dulbecco’s Modified Eagle Media (Gibco, USA) supplemented with 10% fetal bovine serum (Gibco, USA). MDA-MB-436 cells were grown in Leibovitz’s L15 (Gibco, USA) supplemented with 10% fetal bovine serum (Gibco, USA). All cells were cultured in a 5% CO2-humidified atmosphere at 37 °C.

### Construction of Plasmids

FOXM1 DBD cDNA (225–355 aa) was PCR amplified from pCMV-FOXM1 full length plasmid^36^ with primers containing BamHI and EcoRI restriction sites (sense primers: 5′-GCG GAT CCC CAT CAG CGT CCT GGC AGA A-3′ and antisense primers: 5′-GC GAA TTC CTA CAG TGG CTT CAT CTT CCG-3′) and ligated into the pGEX-4T-1 vector. FOXM1 FL (full length) cDNA was PCR amplified from pCMV-FOXM1 plasmid with primers containing BamHI and XbaI restriction sites and the Shine-Dalgarno sequence (AGGAGG) before the ATG (sense primers: 5′-GCT CTA GAA AAG GAG GAC AGC TAT GAA AAC TAG CCC CCG TCG GC-3′ and antisense primers: 5′-GCG GAT CCC TAG TGA TGG TGA TGG TGA TGC TGT AGC TCA GGA ATA A-3′) and ligated into the pET-15b vector. FOXA2 FL (full length) cDNA was PCR amplified from pCMV-FOXA2 plasmid^49^ with primers containing XbaI and BamHI restriction sites and the Shine-Dalgarno sequence (AGGAGG) before the AUG (sense primers: 5′-GCT CTA GAA AAG GAG GAC AGC TAT GCA CTC GGC TTC CAG TAT-3′ and antisense primers: 5′-GCG GAT CCC TAG TGA TGG TGA TGG TGA TGA GAG GAG TTC ATA ATG GG-3′) and ligated into the pET-15b vector. FOXM1 cDNA was PCR amplified from pCMV-FOXM1 plasmid with primers containing BamHI and XbaI restriction sites (sense primers: 5′-GCG GAT CCA TGA AAA CTA GCC CCC GTC GGC-3′ and antisense primers: 5′-GCT CTA GAC TAC TGT AGC TCA GGA ATA A-3′) and ligated into the pEGFP-C2 vector. FOXA2 cDNA was PCR amplified from pCMV-FOXA2 plasmid with primers containing EcoRI and BamHI restriction sites (sense primers: 5′-GGA ATT CAT GCA CTC GGC TTC CAG TAT-3′ and antisense primers: 5′-CGG GAT CCT TAA GAG GAG TTC ATA ATG-3′) and ligated into the pEGFP-C2 vector. The p6xFOXM1 Binding-Luc report plasmid was constructed by ligating a DNA fragment (NheI 5′-GCG CGC TAG CTT TGT TTA TTT GTT TGT TTA TTT GTT TGT TTA TTT GAA GCT TGC GC-3′ HindIII) into the pGL3 basic Luciferase vector (Promega, USA).

### Expression and Purification of Recombinant Proteins

Certain plasmids were transformed into Rosetta/DE3 E. Coli cells and positive colonies were confirmed by colony PCR screening. Cells were grown at 37 °C in LB media until reaching an optical density (OD_600_) of 0.8 and protein expression was induced by addition of 1 mM IPTG for additional 6 hr at 37 °C. The GST protein and GST-FOXM1 DBD protein were purified by Glutathione Sepharose^TM^ 4B (GE, USA) following the manufacturer’s instructions. The FOXM1 FL protein and FOXA2 FL protein were purified by Ni-Sepharose^TM^ 6 Fast Flow (GE, USA) following the manufacturer’s instructions.

### Aptamer Selection by SELEX

Single-stranded DNA (ssDNA) aptamers recognizing FOXM1 DBD were identified using the PCR–based SELEX method^17^. Briefly, a synthetic ssDNA library, containing random 42 nucleotides flanked by 20 nt at each end (5′-AG CAA TGG TAC GGT ACT TCC-42N-GTG CCA CGC TAC TTT GCT AA-3′), was synthesized by Sangon (Shanghai) Co., Ltd, China. The ssDNA library (100 μM, 20 μl, ~2.14 × 10^15^ sequences) was mixed with 500 μl binding buffer (20 mM Hepes, 120 mM NaCl, 5 mM KCl, 1 mM CaCl_2_, 1 mM MgCl_2_, pH 7.35) and heated at 95 °C for 5 min and snap-cooled on ice. GST-FOXM1 DBD protein (10 μg) and Glutathione SepharoseTM 4B beads (50 μl, GE, USA) were added and mixed thoroughly and incubated on ice for 1 hr in a rotary shaker. The mixture was centrifuged at 8000 rpm for 5 min at 4 °C and the pellets was washed twice with binding buffer. The pellets were resuspended with 500 μl binding buffer, heated at 95 °C for 10 min, centrifuged at 13,100 g for 5 min. The collected supernatant was mixed with GST protein (10 μg) and Glutathione SepharoseTM 4B beads (50 μl, GE, USA) and incubated on ice for 1 hr in a rotary shaker. The mixture was centrifuged at 8000 rpm for 5 min at 4 °C to remove the pellet containing non-specific binding ssDNAs. The supernatant was collected and amplified by PCR with the primers (forward: 5′-ROX-A GCA ATG GTA CGG TAC TTC C-3′ and reverse: 5′-Biotin-T TAG CAA AGT AGC GTG GCA C-3′). The PCR products were incubated with streptavidin agarose beads (GE, USA) for 30 min at room temperature and washed 3 times with PBS. The biotin-labeled reverse strand of DNA products were eluted by adding NaOH (1.5 M) and considered as the selected ssDNA pool for the next round of SELEX. After the 6 rounds of selection, the enriched PCR products were cloned with TOPO TA cloning kit (Invitrogen, USA) and the colonies were selected for DNA sequencing.

### Electrophoretic Mobility Shift Assays (EMSA)

The double-strand DNA (dsDNA) probe and aptamers were synthesized by Sangon (Shanghai) Co., Ltd, China, based on the following sequence: dsDNA probe: forward strand 5′-FAM-TTT GTT TAT TTG TTT GTT TAT TTG-3′ (Hot) or 5′-TTT GTT TAT TTG TTT GTT TAT TTG-3′ (Cold), and reverse strand 5′-CAA ATA AAC AAA CAA ATA AAC AAA-3′. FOXM1 Apt: 5′-ROX-TCA CTT CAC ACC GCA TCT CTA CGT CCG GTT GCG CTT TCC TTT-3′ (Hot) or 5′-TCA CTT CAC ACC GCA TCT CTA CGT CCG GTT GCG CTT TCC TTT-3′ (Cold). Control Apt: 5′-ROX-TCA CTT AAA AAA AAA AAA CTC ATG AAA AAA AAA AAA TCC TTT-3′ (Hot) or 5′-TCA CTT AAA AAA AAA AAA CTC ATG AAA AAA AAA AAA TCC TTT-3′ (Cold). FOXM1 Apt (1–15): 5′-ROX-TCA CTT CAC ACC GCA-3′ (Hot) or 5′-TCA CTT CAC ACC GCA-3′ (Cold). FOXM1 Apt (11–34): 5′-ROX-CCG CAT CTC TAC GTC CGG TTG CGC-3′ (Hot) or 5′-CCG CAT CTC TAC GTC CGG TTG CGC-3′ (Cold). FOXM1 Apt (28–42): 5′-ROX-GTT GCG CTT TCC TTT-3′ (Hot) or 5′-GTT GCG CTT TCC TTT-3′ (Cold). Proteins (0.5 mg/ml) were incubated with the FAM-labeled dsDNA probe (50 nM) or ROX-aptamers (50 nM) in binding buffer (20 mM Tris-Cl, 50 mM KCl, 10% glycerol, 0.5 mM EDTA, 0.2 mM DTT, pH 7.6) for 30 min on ice. The dosage of cold probe or aptamers for competitive experiments was in the 100X molar excess. The reactions were resolved in 4% native polyacrylamide gel electrophoresis in 0.5X TBE and visualized with Kodak 4000 MM Imaging System (Kodak, USA) (EX: 465 nm, EM: 535 nm for FAM and EX: 535 nm, EM: 605 nm for ROX).

### Fluorescence Polarization (FP) Assays

Proteins at different concentration were incubated with 10 nM of the FAM-labeled dsDNA probe or ROX-labeled aptamers in binding buffer (20 mM Tris-Cl, 50 mM KCl, 10% glycerol, 0.5 mM EDTA, 0.2 mM DTT, pH 7.6) for 30 min on ice. The FP value of samples were read on Fluoromax-4NIR (Horiba, France) with the EX405 nM/EM535 nM filters for FAM or EX535 nM/EM600 nM filters for ROX in cuvettes.

### Imaging with Confocal Laser-Scanning Microscopes

293T cells (2 × 10^5^ cells in a 60 mm plate) were transiently transfected by Lipofectmin 2000 (Invitrogen, USA) according to the manufacturer’s instructions with pCMV-FOXM1-GFP (5 μg) or pCMV-FOXA2-GFP (5 μg) and 24 hr later were transfected with M-FOXM1 Apt-F (1 μM) or M-Control Apt-F (1 μM) separately. The localization of aptamers and FOXM1 or FOXA2 were imaged with a confocal imaging system (FluoView FV1000, Olympus, Japan), with the ROX fluorescent dye and GFP fluorescent proteins activated by laser at 559 nm and 488 nm respectively.

### Luciferase Activity Assays

293T cells (2 × 10^5^ cells per well in a 6-well plate) were cotransfected with luciferase reporter constructs (p6xFOXM1 Binding-Luc or pCdc25B promoter-Luc) (1 μg) and pCMV-FOXM1 (1 μg). The pRL-CMV plasmid (20 ng) was used as the loading control for each transfection. Control Apt (500 nM) or FOXM1 Apt (100, 300, 500 nM) was also added to certain transfections to test the effects of the aptamers. The luciferase enzyme activities were measured 48 h later with the Dual-Luciferase Assay System (Promega, USA) following the manufacturer’s instructions.

### Pull-Down Assays

Biotinylated aptamers (5 μM) were incubated overnight with cell extracts (500 μg) at 4 °C by end-to-end rotation. Streptavidin agarose beads (20 μL, GE, USA) were applied to samples for 2 hr incubation and the beads were washed three times in lysis buffer (50 mM Tris-Cl, 100 mM NaCl, 2.5 mM EDTA, 2.5 mM EGTA, 1% NP-40, 5% Glycerol). Samples were separated by PAGE and transferred onto PVDF membrane (Millipore, USA). The membrane was blocked with 5% milk and Western Blotting were performed to detect target proteins.

### Isolation RNA, Reverse Transcription and Quantitative Real-time PCR

The total RNA was isolated by Total RNA Kit (Omega, USA) according to the manufacturer’s protocols. The cDNAs were synthesized with M-MLV Reverse Transcriptase (Promega, USA) from total RNA samples. qPCR was performed with SYBR Green (Toyobo, Japan) with following sense (S) and antisense (AS) primers: hFOXM1-S, 5′-GCT TGC CAG AGT CCT TTT TGC-3′ and hFOXM1-AS, 5′-CCA CCT GAG TTC TCG TCA ATG C-3′; hCdc25B-S, 5′-AGT CCT GAC CGG AAG ATG GA-3′ and hCdc25B-AS, 5′-GAT GTT GCT GAA CTT GCC CG-3′; hCyclinB1-S, 5′-GGT CTG GGT CGG CCT CTA CCT-3′ and hCyclinB1-AS, 5′-AGC CAG GTG CTG CAT AAC TGG AA-3′; hGAPDH-S, 5′-GGA GCG AGA TCC CTC CAA AAT-3′ and hGAPDH-AS, 5′-GGC TGT TGT CAT ACT TCT CAT GG-3′. The qPCR was performed in the realplex2 qPCR system (Eppendorf, Germany).

### Western Blot Assays

Whole-cell lysates were separated using SDS-PAGE and transferred onto PVDF membrane for western blotting. The following antibodies and dilutions were used for Western blotting: rabbit anti-FOXM1 (1:5000, SantaCruz sc-502), rabbit anti-Cdc25B (1:1000, SantaCruz sc-326), mouse anti-CyclinB1 (1:1000, SantaCruz sc-166757), mouse anti-β-actin (1:10000, Abcam ab49900). The signals from the primary antibody were amplified by horseradish peroxidase (HRP) conjugated anti-rabbit IgG (1:10000, GE LNA934VAE) or anti-mouse IgG (1: 10000; BioRad 170–6516), and detected with Super Signal West Femto Maximum Sensitivity Substrate (Thermo, USA) by Kodak 4000 MM Imaging System (Kodak, USA).

### Circular Dichroism (CD) Measurement

Aptamers (5 μM) were resuspended in binding buffer (20 mM Tris-Cl, 50 mM KCl, 10% glycerol, 0.5 mM EDTA, 0.2 mM DTT, pH 7.6), heated at 95◦C for 5 min, and snap-cooled on ice. CD spectra were collected on a MOS-500 Spectrometer (Biologic, France) equipped at 200–350 nm, 0.2 s Acq duration, 4 nm Fentes bandwidth.

### Flow cytometry analysis of cell cycle

The tested cells were collected and washed twice with cold PBS. Cells were fixed overnight in 70% ethanol at 4°C and collected by centrifugation 3,000 r.p.m. for 2 min. The cell pellets were resuspended in propidium iodide (0.05 mg/ml) plus RNase (0.02 mg/ml) and incubated in the dark at room temperature for 30 min. The cells were filtered and analyzed for DNA content on a QuantaSC flow cytometer (Beckman, USA).

### Statistical Analysis

The experimental data are expressed as the mean ± standard deviation. The comparison among groups was performed using one-way analysis of variance and Dunnett-t tests were used for comparison between experimental groups and control groups. P < 0.05 was considered to indicate a statistically significant difference.

## Additional Information

**How to cite this article**: Xiang, Q. *et al*. Suppression of FOXM1 Transcriptional Activities via a Single-Stranded DNA Aptamer Generated by SELEX. *Sci. Rep.* **7**, 45377; doi: 10.1038/srep45377 (2017).

**Publisher's note:** Springer Nature remains neutral with regard to jurisdictional claims in published maps and institutional affiliations.

## Supplementary Material

Supplementary Information

## Figures and Tables

**Table 1:** The sequences of selected colonies (n = 40) from the sixth round of SELEX.

Clone NO.	Sequence (5′ → 3′)	Copies	Percentage
C1 (FOXM1 Apt)	TCA CTT CAC ACC GCA TCT CTA CGT CCG GTT GCG CTT TCC TTT	17	42.5%
C2	TCC CAG TCA CGA CGT TGT AAA ACG ACG GCC AGT GAA TTG TAG	8	20%
C3	CTG AAA GCG CAA CCG GAC GTA TAC AAG CGG GGG TCC TTG ATT	6	15%
C4	TTA CTG GAG CCC CGC ATC ACT GCG ATC CGG GTG CGG TTC CCT	6	15%
Other sequences			7.5%
