# Supplementary material for: Suppression of FOXM1 Transcriptional Activities via a Single-Stranded DNA Aptamer Generated by SELEX
Source: Sci Rep. 2017 Mar 30;7:45377. doi: 10.1038/srep45377 (PMC5371818; doi:10.1038/srep45377)
Supplement: Supplementary Information [file srep45377-s1.pdf]

Suppression of FOXM1 Transcriptional Activities via a Single-Stranded DNA Aptamer Generated by SELEX

Qin Xiang, Guixiang Tan*, Xia Jiang, Kuangpei Wu, Weihong Tan, and Yongjun Tan*

Supplementary Figures (Figure S1-S9)

Figure S1

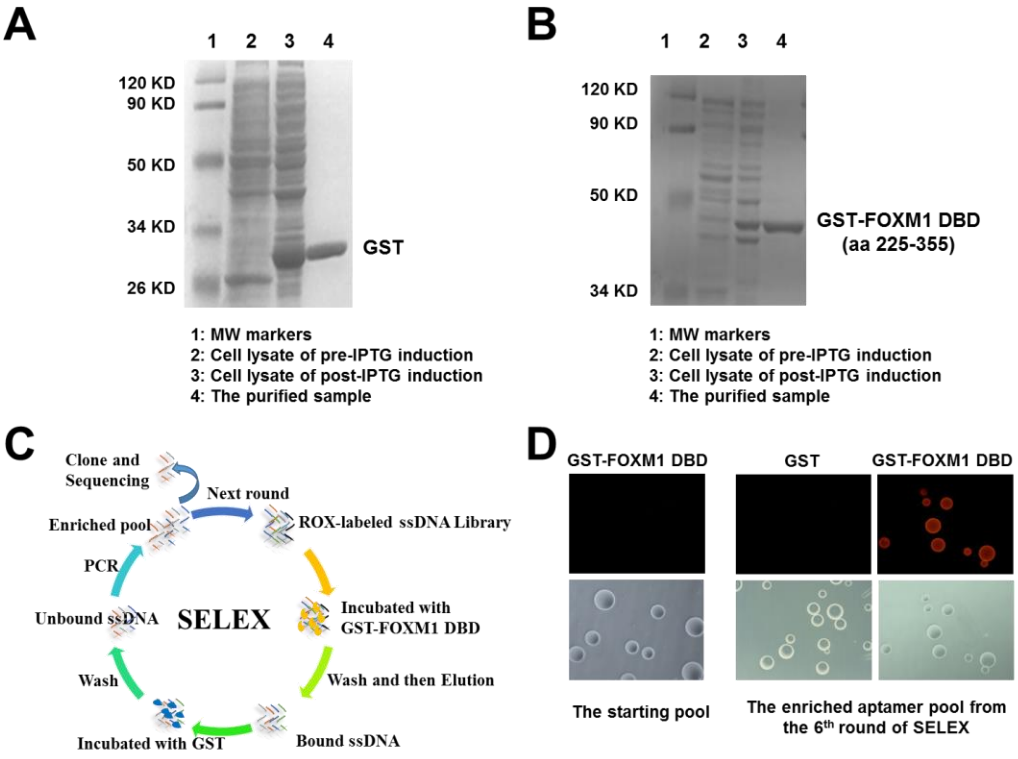

In vitro selection of FOXM1 DBD-specific aptamers by SELEX. (A-B) The purification of GST proteins and GST-FOXM1 DBD fusion proteins. The pGEX-4T-1 plasmids expressing GST protein or the pGEX-4T-1-FOXM1-cDNA-fragment plasmids expressing GST-FOXM1 DBD (225-355 aa) fusion protein were transformed into Rosetta/DE3 E. coli cells. The cells were grown at 37˚C in LB media to optical density (OD_{600} ~ 0.8) and added with IPTG (final concentration 1 mM) to induce protein expression for additional 6 hr culture. The GST (A) or GST-FOXM1 DBD fusion proteins (B) were purified by Glutathione Sepharose^{TM} 4B beads, separated by PAGE, and stained with Commassie Blue. (C) The schematic SELEX procedure for FOXM1 DBD-specific aptamer selection. The FOXM1 DBD-specific aptamers were selected from a carboxy-X-rhodamine (ROX)-labeled ssDNA library according to the SELEX procedure described in Materials and Methods in detail. (D) The FOXM1 DBD-specific aptamers were enriched after the 6th round of SELEX. The enriched aptamer pools from the 6th round of SELEX were incubated with GST or GST-FOXM1 DBD fusion protein Glutathione Sepharose beads and observed with fluorescence microscope (100x). Briefly, the amplified PCR products from 6th round of SELEX were incubated with streptavidin agarose beads for 30 min at room temperature, washed 3 times with PBS, and eluted by adding NaOH (1.5 M) to generate the biotin-labeled single strand DNA products. These products were mixed with 500 μl binding buffer and heated at 95° C for 5 min and snap-cooled on ice. GST or GST-FOXM1 DBD protein (10 μg) and Glutathione SepharoseTM 4B beads (50 μl) were added and mixed thoroughly and incubated on ice for 1 hr in a rotary shaker. The mixture was centrifuged at 8000 rpm for 5 min at 4° C and the pellets were washed twice with binding buffer. The re-suspended samples were observed under fluorescence microscope. The starting pool was also incubated with GST-FOXM1 DBD fusion protein Glutathione Sepharose beads as a negative control.

Figure S2

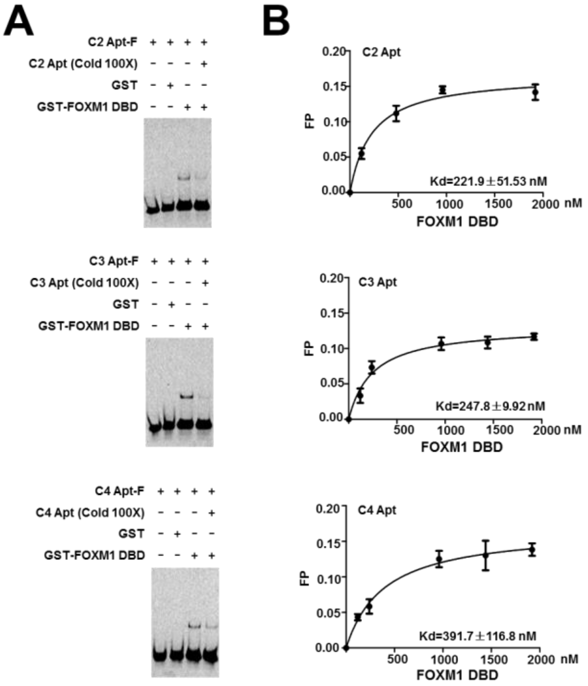

Identification of the binding ability and affinity of other cloned aptamers to FOXM1 DBD. (A) The ROX-labeled C2, C3, or C4 aptamer (C2 Apt-F, C3 Apt-F, C4 Apt-F, 50 nM each) was used for EMSA experiments with the purified GST or GST-FOXM1 DBD fusion proteins (0.5 mg/ml). The unlabeled aptamer (C2 Apt, C3 Apt, or C4 Apt, 100x) was added to certain reactions to show specificity of aptamer/GST-FOXM1 DBD complex formation. GST proteins were used as the negative controls in the assays. (B) FP assays were performed with C2 Apt-F, C3 Apt-F, or C4 Apt-F (10 nM each sample) plus the FOXM1 DBD protein at different concentration (0, 120, 240, 960, 1440, 1920 nM). The FP values were obtained with the EX535nm/EM600nm filter for ROX in Fluoromax-4NIR (Horiba).

Figure S3

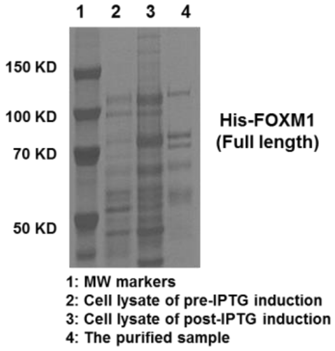

The purification of FOXM1 full-length protein. The pET-15b-FOXM1-cDNA plasmids were transformed into Rosetta/DE3 E. coli cells. The cells were grown at 37˚C in LB media to optical density (OD_{600} ~ 0.8) and added with IPTG (final concentration 1 mM) to induce protein expression for additional 6 hr culture. The His-FOXM1 full-length protein were purified by Ni-SepharoseTM 6 Fast Flow, separated by PAGE, and stained with Commassie Blue.

Figure S4

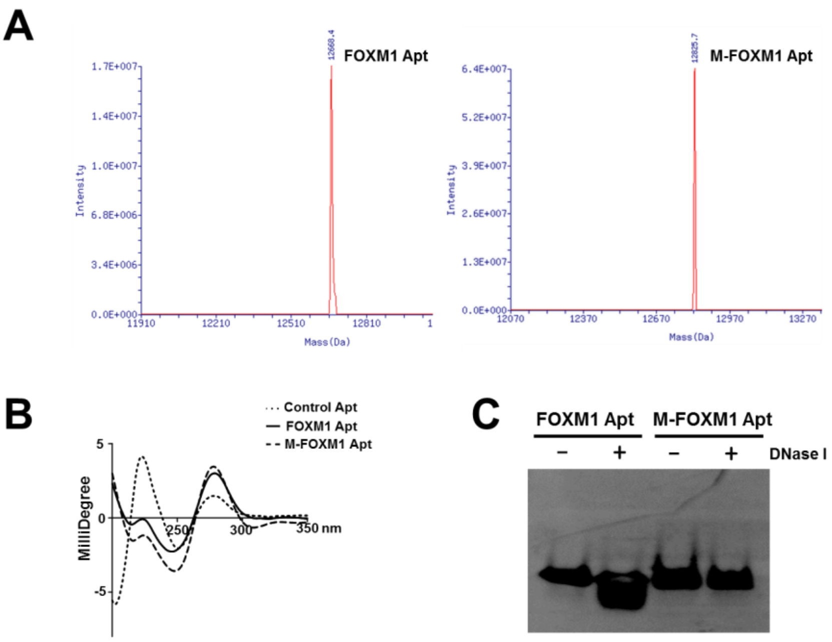

The comparison between FOXM1 Apt and M-FOXM1 Apt. (A) Mass Spectrometry (MS) analysis of FOXM1 Apt and M-FOXM1 Apt. The molecular mass of M-FOXM1 Apt was higher than that of FOXM1 Apt because of the phosphorthioate modification. (B) Circular Dichroism (CD) analysis of Control Apt, FOXM1 Apt, and M-FOXM1 Apt. Control Apt, FOXM1 Apt, and M-FOXM1 Apt (5 μM) were analyzed in CD spectra (MOS-500 Spectrometer) at 200-350 nm, 0.2 s Acq duration, 4 nm Fentes bandwidth. (C) The DNase I treatment of FOXM1 Apt and M-FOXM1 Apt. FAM-labeled FOXM1 Apt and M-FOXM1 Apt (1 pM) were treated with DNase I (1 U) for 30 min. The samples were separated by PAGE and visualized with Kodak 4000MM Imaging System (EX: 465 nm, EM: 535nm).

Figure S5

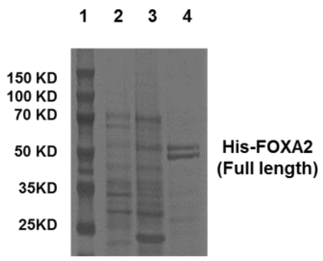

1: MW markers
2: Cell lysate of pre-IPTG induction
3: Cell lysate of post-IPTG induction
4: The purified sample

The purification of FOXA2 full-length protein. The pET-15b-FOXA2-cDNA plasmids were transformed into Rosetta/DE3 E. coli cells. The cells were grown at 37˚C in LB media to optical density (OD_{600} ~ 0.8) and added with IPTG (final concentration 1 mM) to induce protein expression for additional 6 hr culture. The GST-FOXA2 full-length protein were purified by Ni-SepharoseTM 6 Fast Flow, separated by PAGE, and stained with Commassie Blue.

Figure S6

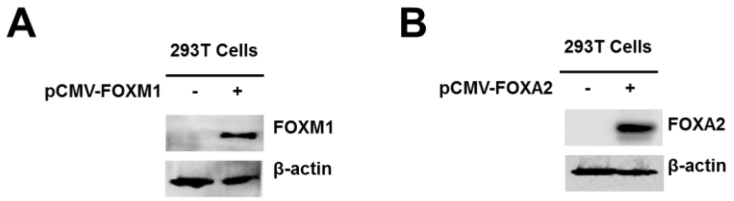

The preparation of FOXM1-overexpressed and FOXA2-overexpressed cell lyates. HEK293T cells were transfected with pCMV-FOXM1 or pCMV-FOXA2 expression vectors (5 μg) and 48 hr later the whole-cell lysates were prepared. The overexpression of FOXM1 (A) or FOXA2 (B) were confirmed by Western Blotting with anti-FOXM1 or anti-FOXA2 antibody. The levels of β-actin were measured as loading controls by Western Blotting.

Figure S7

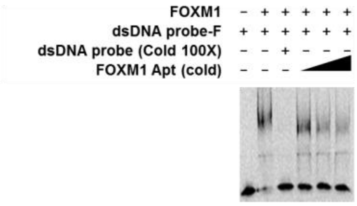

FOXM1 Apt abolished the binding ability of FOXM1 to its putative DNA binding sequence. The dsDNA probe-F (50 nM each sample) was mixed with His-FOXM1 proteins (0.5 mg/ml) for EMSA experiments. The FOXM1 Apt (unlabeled) was added to the reactions at increased concentration (500, 2500, 5000 nM) to act as the competitor of FOXM1/DNA binding. The unlabeled dsDNA probe (100x) was used to show specificity of FOXM1/DNA complex formation.

Figure S8

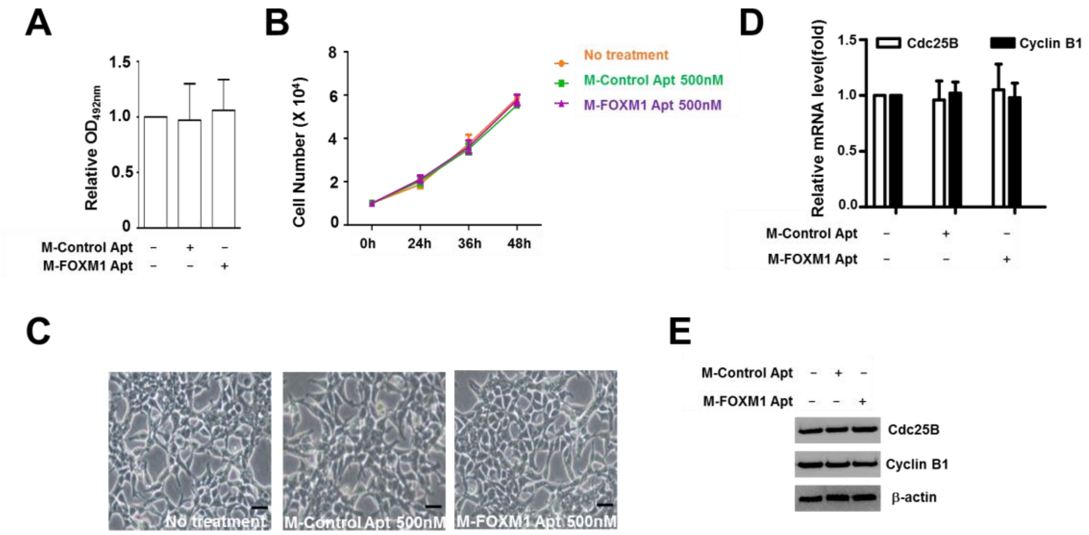

The analysis of the FOXM1 Apt effects on the wild type 293T cells. (A) The 293T cells (1 x 10^3 cells/well in triplicates) were seeded in each well of the 96-well tissue culture plates and 12 hr later were transfected with M-Control Apt or M-FOXM1 Apt (500 nM). MTT measurements were performed 48 hr later. (B) The growth curve of M-FOXM1 Apt-treated 293T cells. The 293T cells (1 x 10^4 cells/well in triplicates) were seeded in each well of the six-well tissue culture plates and 12 hr later were transfected with M-Control Apt or M-FOXM1 Apt (500 nM). The cell numbers in each well were counted at different time points (24 hr, 36 hr, 48 hr) post transfection. (C) The morphology of 293T cells post M-FOXM1 Apt treatment. The 293T-FOXM1 cells were transfected with M-Control Apt or M-FOXM1 Apt (500 nM) and 48 hr later the cells were imaged with the microscope (TE-2500, NIKON) (100x). (D-E) The 293T cells were transfected with M-Control Apt or M-FOXM1 Apt (500 nM) and 48 hr later the cell samples were harvested for the preparation of total RNA and total proteins. The levels of Cdc25B and Cyclin B1 in the cells were examined by qPCR for mRNA levels (D) and by Western blotting for protein levels (E).

Figure S9

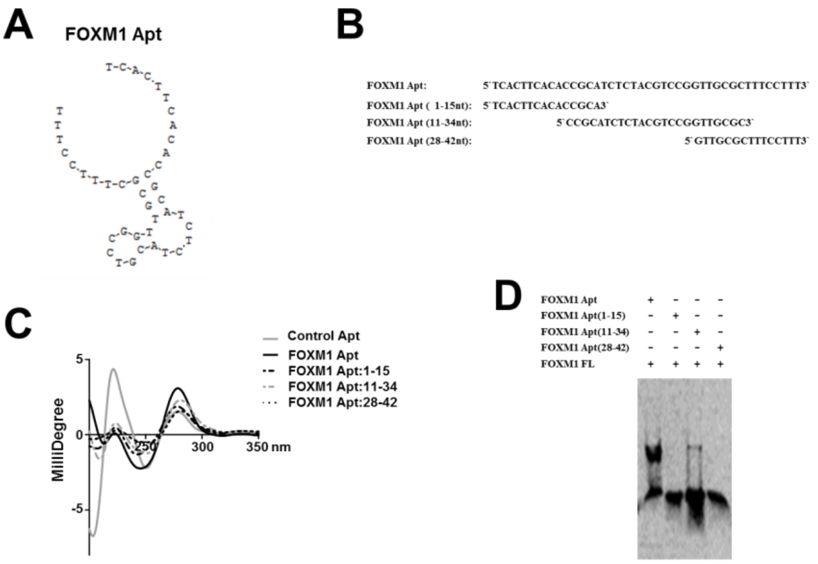

Identification of the core FOXM1-binding sequence in FOXM1 Apt. (A) The DNAMAN software was used to predict the secondary structure of FOXM1 Apt, which had a primarily stem-loop-loop structure. (B) The schematic of three truncated sequences of FOXM1 Apt (FOXM1 Apt (1-15), FOXM1 Apt (11-34), and FOXM1 Apt (28-42)). (C) CD analysis of the truncated FOXM1 Apt sequences (5 μM) in CD spectra at 200-350 nm, 0.2 s Acq duration, 4 nm Fentes bandwidth. (D) The truncated FOXM1 Apt (11-34) possessed a decreased binding activity to FOXM1 in the EMSA experiment. FOXM1 Apt-F and different ROX-labeled truncated FOXM1 Apts (FOXM1 Apt (1-15)-F, FOXM1 Apt (11-34)-F, and FOXM1 Apt (28-42)-F) (50 nM) were used for EMSA experiments with the purified His-FOXM1 full-length protein (FOXM1) (0.5 mg/ml). The electrophoresis was performed and the DNA probe/protein complexes were visualized by fluorescence imaging.
